# Predicting mucosal healing in patients with ulcerative colitis in clinical remission using biomarkers: A single-center prospective pilot study

**DOI:** 10.1097/MD.0000000000047536

**Published:** 2026-02-13

**Authors:** Naohiro Kato, Ryusaku Kusunoki, Hiroki Kamada, Shigeaki Semba, Yuji Teraoka, Takeshi Mizumoto, Yuzuru Tamaru, Tsuyoshi Hatakeyama, Atsushi Yamaguchi, Hirotaka Kouno, Shigeto Yoshida, Toshio Kuwai

**Affiliations:** a Department of Endoscopy, NHO Kure Medical Center and Chugoku Cancer Center, Kure, Japan; b Department of Gastroenterology, NHO Higashi Hiroshima Medical Center, Higashi Hiroshima, Japan; c Department of Gastroenterology, NHO Kure Medical Center and Chugoku Cancer Center, Kure, Japan; d Gastrointestinal Endoscopy and Medicine, Hiroshima University Hospital, Hiroshima, Japan; e Institute for Clinical Research, NHO Kure Medical Center and Chugoku Cancer Center, Kure, Japan.

**Keywords:** biomarkers, clinical remission, mucosal healing, ulcerative colitis

## Abstract

Treating ulcerative colitis (UC) requires mucosal healing (MH); however, clinical remission does not always involve MH. Fecal calprotectin (FC) is a useful marker to determine MH. Leucine-rich alpha-2 glycoprotein (LRG) and prostaglandin E-major urinary metabolite (PGE-MUM) have similar performance to FC and may also predict MH. No previous studies have provided a detailed comparative analysis of LRG, PGE-MUM, and FC. Herein, we investigated their associations with endoscopic activity and their potentials as predictors of MH. This single-center, prospective, observational study included patients with ulcerative colitis in clinical remission for >3 months (partial Mayo score ≤ 2) who were to undergo colonoscopy between July 2023 and June 2024. Endoscopic remission (ER) was defined as Mayo endoscopic score of 0, while histological remission (HR) was based on the Geboes score. Overall, 46 patients were enrolled, and all underwent colonoscopy; 20 (43%) had ER, and 9 (20%) had HR. The median LRG, PGE-MUM, and FC levels were significantly higher in patients without ER than in those who achieved ER (*P* < .05). The areas under the receiver operating characteristic curves of LRG, PGE-MUM, and FC for determining ER were 0.686 (95% confidence interval [CI]: 0.530–0.845), 0.695 (95% CI: 0.552–0.872), and 0.788 (95% CI: 0.658–0.919), respectively. The optimal cutoff value obtained from the receiver operating characteristic curve, LRG, PGE-MUM, and FC values for determining ER were 14.2 µg/mL, 30.6 μg/gCr, and 143 mg/kg, respectively. The areas under the receiver operating characteristic curves for LRG + FC and PGE-MUM + FC to determine ER were 0.800 (95% CI: 0.672–0.928) and 0.865 (95% CI: 0.764–0.966), respectively. LRG and PGE-MUM are potential biomarkers for determining ER in clinical remission. Combining LRG and PGE-MUM assessments with FC may improve the accuracy of confirming ER in ulcerative colitis, even during the remission phase.

## 1. Introduction

Ulcerative colitis (UC) is a chronic idiopathic intestinal disorder and is the most common inflammatory bowel disease (IBD) in Europe and the USA as well as in Asia.^[1]^ The activity of UC-associated decline in the quality of life, and recurring inflammation can result in colon cancer.^[2]^ Studies have emphasized the need to look beyond symptoms and to treat endoscopic/macroscopic lesions in UC, with the aim of preventing structural damage and disability.^[3]^ Managing UC requires continuous treatment and appropriate assessment of disease activity, including clinical, endoscopic remission (ER), and histological remission (HR). Mucosal healing (MH), which is assessed by endoscopy, is known as deep remission and is associated with lower recurrence rates and a favorable prognosis.^[4,5]^ Imaging tests, specifically endoscopy, are the gold standard for evaluating disease activity; however, discrepancy between clinical symptoms and endoscopic findings is common in clinical practice. Additionally, it is important to employ noninvasive and repeatable biomarkers to confirm MH as clinical remission does not always indicate MH.^[6]^ Therefore, we need to define the treatment target and frequency of assessment to reach this target.^[7]^

Several biomarkers that reflect the disease activity in patients with IBD have been developed, including fecal markers. Fecal calprotectin (FC) is reportedly correlated with the disease activity of IBD,^[8,9]^ and it was proposed as a fecal marker for bowel inflammation. However, measuring FC has disadvantages, including the absence of an absolute cutoff value to confirm remission, individual variations in remission line, and long turnaround times to obtain results. Additionally, patients must collect their feces and bring them to the hospital for both fecal immunochemical test and FC. Furthermore, FC levels have a wide positive range and do not necessarily exhibit a strong correlation with the endoscopic activity score.^[10]^

Leucine-rich alpha-2 glycoprotein (LRG) is a novel biomarker for several diseases, including rheumatoid arthritis.^[11]^ Serum LRG levels are reportedly elevated in autoimmune diseases and correlated with the disease activity in rheumatoid arthritis, systemic lupus erythematosus, adult-onset Still’s disease, systemic juvenile idiopathic arthritis, primary biliary cholangitis, and IBD.^[12]^ Levels of serum LRG reportedly have a good correlation with MH and the disease activity of UC, and it is included in the insurance coverage in Japan as of 2020.^[13–15]^

Prostaglandin E-major urinary metabolite (PGE-MUM) is excreted in the urine as a metabolite of prostaglandin E2. PGE-MUM levels are reportedly associated with the extent of colonic inflammation in UC^[16]^ and are strongly correlated with Mayo endoscopic subscore (MES) and Matts’ grading. Therefore, they may be used to confirm MH in UC.^[17]^ Ishida et al examined the usefulness of PGE-MUM in predicting relapse in patients with UC during remission.^[18]^ They reported that the clinical relapse prediction ability of PGE-MUM was higher than that of the endoscopic score (MES 0 vs MES 1). An advantage of PGE-MUM is that it can be easily and noninvasively measured even in small amounts of urine samples. Hagiwara reported the high predictive accuracy for MES 0, with PGE-MUM showing similar performance to FC.^[19]^ In Japan, PGE-MUM was covered by insurance in 2024, and its future applications are highly anticipated.

During flare-ups of UC, biomarkers are not necessary as UC activity can be easily evaluated. A truly significant biomarker is 1 that can identify the presence of endoscopic activity during clinical remission. LRG and PGE-MUM are correlated with the endoscopic activity and are able to predict MH in clinical practice in patients with UC; however, their performance was not superior to that of fecal markers, and these studies have not conducted a detailed sensitivity analysis comparing LRG, PGE-MUM, and FC at the same time for predicting MH in UC in clinical remission.^[15,17]^ Therefore, this study focused on clinical remission and aimed to investigate the association of LRG, PGE-MUM, and FC at the same time with the endoscopic activity of UC and their predictive ability for MH compared with other biomarkers.

## 2. Methods

### 2.1. IRB/IACUC approval

This study conformed to the principles of the Declaration of Helsinki. Ethical approval was obtained from the Ethics Committee of National Hospital Organization Kure Medical Center and Chugoku Cancer Center (approval no.: 2023-08). Informed consent was obtained from all patients included in the study.

### 2.2. Description of participants

We conducted a prospective observational study at the National Hospital Organization Kure medical center in Hiroshima, Japan. Patients were included if they were diagnosed with UC, in clinical remission for >3 months (partial Mayo score ≤ 2), and scheduled to undergo colonoscopy between July 2023 and June 2024. The inclusion criteria for combination therapy required that patients have been on a stable dose of specific treatments before the trial began. Specifically, patients were treated with 5-aminosalicylic acid for at least 4 weeks, with immunomodulators (e.g., azathioprine and mercaptopurine) for at least 4 weeks, or with biological agents (e.g., tumor necrosis factor inhibitors) for at least 8 weeks before the trial. Furthermore, written informed consent must be obtained from the patient or their legally authorized representative. The exclusion criteria included patients who were hospitalized, those with an artificial anus, pregnant patients or those who may be pregnant, those with inflammatory diseases other than UC (e.g., Crohn’s disease), and those who have received treatments, including nonsteroidal anti-inflammatory drugs or aspirin, budesonide enemas, corticosteroids, or blood component removal therapies, within 4 weeks prior to the trial, and those with untreated malignancies.

### 2.3. Study design

This was a single-center prospective pilot study. In this study, ER was defined as an MES of 0. HR was defined based on the Geboes score grade and as follows: no or mild increase in chronic inflammatory infiltrate in the lamina propria, no neutrophils in the lamina propria or epithelium, and no erosion, ulceration, or granulation tissue (Grade 0: ≤0.3, Grade 1: ≤1.1, Grade 2a: ≤2A.3, Grade 2b: 2B.0, Grade 3: 3.0, Grade 4: 4.0, Grade 5: 5.0).^[20]^ For the primary analysis, we analyzed differences in the PGE-MUM, LRG, FC, and fecal immunochemical test values between the 2 groups, which were classified as success or failure in achieving ER and HR in patients with UC in clinical remission. For the secondary analysis, the diagnostic accuracy of PGE-MUM, LRG, FC, and fecal immunochemical test for confirming ER and HR was compared using the area under the curve (AUC) of the receiver operating characteristic (ROC) curves. Additionally, the optimal cutoff values for determining ER and HR were evaluated. Sample size number was selected based on the previous published pilot and feasibility trials with a median sample size of anywhere from 12 per group to 35 per group.^[21,22]^ Because this was a pilot study to predict MH and the disease activity of UC.

### 2.4. Measurement of the biomarkers

FC levels were measured on or before the day of colonoscopy preparation using fluorescence enzyme immunoassay. Participants prepared fecal samples using an EliA Calprotectin kit (Thermo Fisher Diagnostics K.K., Tokyo, Japan). Serum LRG levels were analyzed using a NANOPIA LRG Kit (Sekisui Medical, Tokyo, Japan) based on the latex turbidimetry method and were measured either the day before or the day following colonoscopy. Spot urinary samples to measure PGE-MUM levels were collected either on the day before or after colonoscopy as the laxatives used during the colonoscopy preparation may influence PGE-MUM levels. Values were measured by chemiluminescent enzyme immunoassay (SRL, Tokyo, Japan). PGE-MUM levels were normalized to the creatinine concentration and expressed as μg/gCr.

### 2.5. Colonoscopy

All colonoscopies were performed within 2 weeks of measuring biomarker levels. In all participants, at least 1 biopsy specimen was obtained from each segment of the colon. The endoscopic status of all participants was evaluated according to the MES classification. MES evaluations were performed by expert physicians at each portion of the colorectum (cecum and ascending colon combined, transverse colon, descending colon, sigmoid colon, and rectum), and the maximum score in the colorectum was used for the analysis. The scores were determined following discussion among the 2 experts if the assigned scores differed for the individual patients. Active inflammation was defined as an MES of 2 or 3. Expert pathologists evaluated the Geboes score grade to determine the histological activity of UC. If more than 2 biopsy specimens were obtained, the highest score was used as the Geboes score.

### 2.6. Statistical analysis

For the primary analysis, Wilcoxon rank-sum tests were employed to compare differences in LRG, PGE-MUM, and FC values based on the success or failure in achieving ER and HR. For the secondary analysis, AUCs of the ROC curves for LRG, PGE-MUM, and FC values were calculated to determine ER and HR. ROC analysis was used to compare the AUCs of the 3 markers. AUC comparisons were performed using DeLong’s test. The optimum cutoff was obtained by searching for the value with the maximum Youden index. The combination of biomarkers for evaluating ER was defined as cases that satisfy both cutoff values of the biomarkers. Statistical significance was set at *P* < .05, and data on the baseline and clinical characteristics were expressed as mean ± standard deviation, median (range, interquartile range [IQR]), or percentages. All statistical analyses were performed using JMP Pro 16 (SAS Institute, Cary).

## 3. Results

### 3.1. Patient characteristics

Overall, 46 patients were enrolled in this study. All participants underwent colonoscopy, and blood, urine, stool, and biopsy specimens were obtained. The clinical characteristics of the participants are shown in Table 1. The median age was 63 (45–74) years, and majority had extensive or left-sided colitis (84.8%); proctitis was noted in 15.2% of the patients. The number of patients with ER and HR was 20 (43%) and 9 (20%), respectively, whereas that who achieved ER and HR was 9 (20%).

**Table 1:** Patients’ characteristics.

Patient’s number	n = 46
Age, years, median (IQR)	63 (45–74)
Male sex, n (%)	26 (56.5)
Disease duration (year), median (IQR)	9.7 (3.4–17)
Disease extent, n (%)	
Extensive colitis	26 (56.5)
Left-sided colitis	13 (28.3)
Proctitis	7 (15.2)
MES, n (%)	
MES 0	20 (43.5)
MES 1	12 (26.1)
MES 2	12 (26.1)
MES 3	2 (4.3)
Geboes histopathology score, median (IQR)	
Grade 0	1 (1–2)
Grade 1	2 (1–2)
Grade 2a	1 (0–1)
Grade 2b	1 (0–1)
Grade 3	0 (0–1)
Grade 4	0 (0–1)
Grade 5	1 (0–1)
Concomitant drug, n (%)	
Oral 5-ASA	36 (78.3)
Suppository 5-ASA	7 (15.2)
Suppository steroids	0 (0)
Systemic steroids	0 (0)
Immunomodulators	10 (21.7)
Biologics	13 (28.3)
Treatment duration (month), median (IQR)	
Oral 5-ASA	23 (9.3–44)
Suppository 5-ASA	8.6 (6.8–17)
Immunomodulators	32 (21–57)
Biologics	19 (4.1–25)

5-ASA = 5-aminosalicylic acid, ER = endoscopic remission, IQR = interquartile range, MES = Mayo endoscopic score, UCEIS = ulcerative colitis endoscopic index of severity.

### 3.2. Comparison of biomarkers between the groups

**Figure 1.**
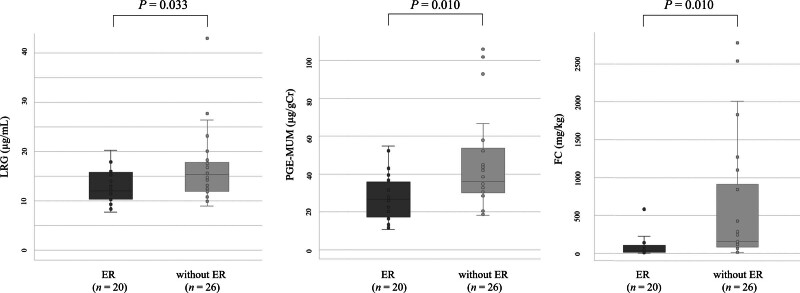
Comparison of leucine-rich alpha-2 glycoprotein (LRG), prostaglandin E-major urinary metabolite (PGE-MUM), and fecal calprotectin (FC) levels between patients with endoscopic remission (ER) and without ER.

**Figure 2.**
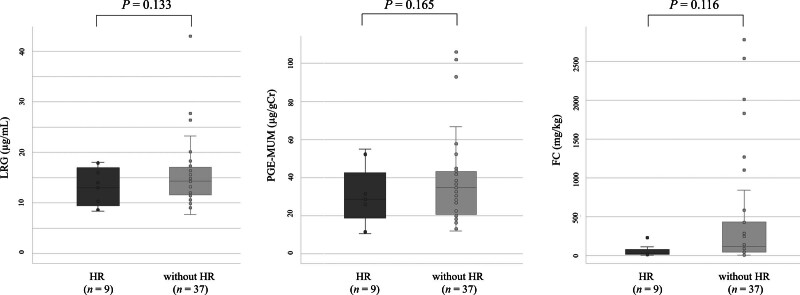
Comparison of leucine-rich alpha-2 glycoprotein (LRG), prostaglandin E-major urinary metabolite (PGE-MUM), and fecal calprotectin (FC) levels between patients with histological remission (HR) and without HR.

As shown in Figure 1, the median LRG, PGE-MUM, and FC levels were significantly lower in patients who achieved ER than in those without ER (12.1 [10.4–15.8] µg/mL vs 15.4 [12.0–17.8] µg/mL [*P* = .033] for LRG, 26.7 [17.4–35.8] µg/gCr vs 36.1 [30.1–53.7] µg/gCr [*P* = .010] for PGE-MUM, and 35.2 [17.1–107] mg/kg vs 160 [85.4–909] mg/kg [*P* = .010] for FC). The LRG, PGE-MUM, and FC levels tended to be lower in patients who achieved HR than in those without HR (13.0 [9.5–17.0] µg/mL vs 14.3 [11.6–17.1] µg/mL [*P* = .133] for LRG, 28.7 [18.8–42.6] µg/gCr vs 34.8 [20.7–43.3] µg/gCr [*P* = .165] for PGE-MUM, and 27.7 [13.4–76.5] mg/kg vs 118 [41.5–430] mg/kg [*P* = .116] for FC) (Fig. 2).

### 3.3. Efficacy of biomarkers for the evaluation of MH in patients with UC in clinical remission

**Figure 3.**
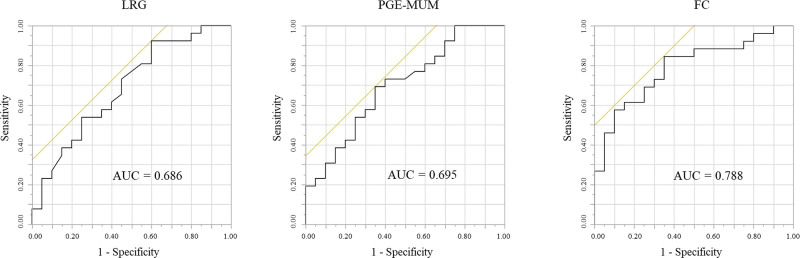
The area under the receiver operating characteristic (ROC) curve for leucine-rich alpha-2 glycoprotein (LRG), prostaglandin E-major urinary metabolite (PGE-MUM), and fecal calprotectin (FC) levels between patients with endoscopic remission (ER) and without ER.

**Figure 4.**
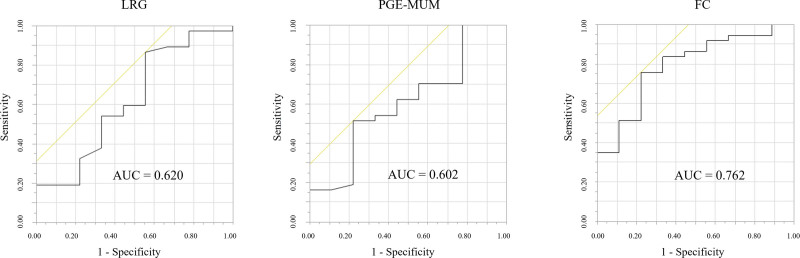
The area under the receiver operating characteristic (ROC) curve for leucine-rich alpha-2 glycoprotein (LRG), prostaglandin E-major urinary metabolite (PGE-MUM), and fecal calprotectin (FC) levels between patients with histological remission (HR) and without HR. AUC = area under the curve.

Given the potential role of LRG and PGE-MUM as biomarkers for endoscopic activity of UC with clinical remission, we investigated their diagnostic accuracy in detecting MH. We compared the sensitivity and specificity of LRG and PGE-MUM with those of FC using ROC curves and AUC analyses in detecting ER. The AUCs of the ROC curves of LRG, PGE-MUM, and FC for confirming ER were 0.686 (95% CI: 0.530–0.845), 0.695 (95% CI: 0.552–0.872), and 0.788 (95% CI: 0.658–0.919) (Fig. 3), and HR were 0.620 (95% CI: 0.393–0.848), 0.602 (95% CI: 0.384–0.820), and 0.762 (95% CI: 0.609–0.926) (Fig. 4), respectively. In this study, the optimal cutoff value obtained from ROC curve, LRG, PGE-MUM, and FC values for determining ER were 14.2 µg/mL, 30.6 μg/gCr, and 143 mg/kg, respectively.

### 3.4. Combination of biomarkers for the evaluation of MH in patients with UC in clinical remission

**Figure 5.**
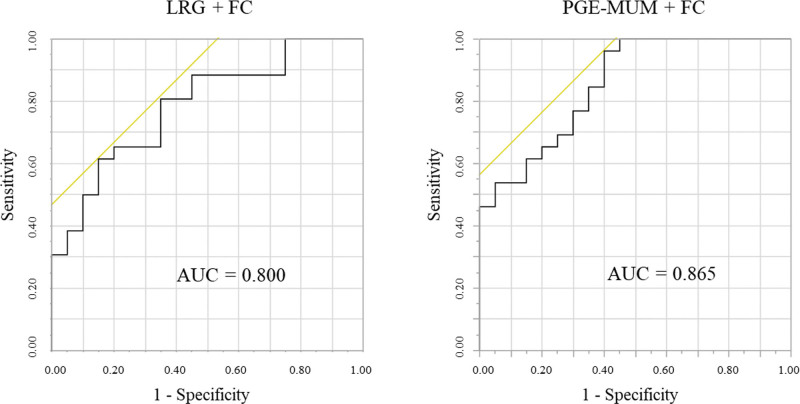
The area under the receiver operating characteristic (ROC) curve for leucine-rich alpha-2 glycoprotein (LRG) combined with fecal calprotectin (FC) versus prostaglandin E-major urinary metabolite (PGE-MUM) combined with FC between patients with endoscopic remission (ER) and without ER.

For FC, the highest AUC result was observed. However, FC has a wide range of values, which can make it difficult to assess MH. In contrast, the combination of blood LRG and urinary PGE-MUM, which have less fluctuation, may be effective biomarkers for MH. Figure 5 shows that FC + LRG and FC + PGE-MUM more accurately confirmed MH. The AUCs of the ROC curves for LRG + FC and PGE-MUM + FC to confirm ER were 0.800 (95% CI: 0.672–0.928) (*P* = .410) and 0.865 (95% CI: 0.764–0.966) (*P* = .265), in comparison with FC alone, respectively. Thus, combining biomarkers allowed for a more accurate evaluation than using single biomarkers.

## 4. Discussion

FC has been useful for predicting MH; however, evaluating fecal biomarkers has disadvantages such as stool sample collection being a psychological burden for patients especially those living in large and crowded cities. Therefore, FC is not reliable despite its high AUC, and its usefulness is limited. Meanwhile, serum or urine sample collection is a less invasive method for evaluating MH. As previously reported, LRG and PGE-MUM are useful for predicting MH and the disease activity of UC. Mucosal conditions may be evaluated using LRG or PGE-MUM instead of regular colonoscopy. LRG and PGE-MUM levels are strongly correlated with UC activity and are comparable to fecal biomarkers in evaluating the mucosal condition of patients with UC; however, these studies mostly focused on patients with active UC and there were no reports have directly compared these biomarkers.^[23]^

Therefore, our study, which focused on patients with UC in remission, is meaningful. Our results revealed that both LRG and PGE-MUM levels were significantly different between patients who achieved and did not achieve ER. Meanwhile, the AUC was similar between LRG and PGE-MUM levels when confirming ER and HR. These results showed that LRG and PGE-MUM are comparable when assessing for MH. Additionally, our results also showed that compared with FC, both LRG and PGE-MUM may confirm ER in patients with UC in clinical remission, although their performance was inferior to that of FC. This is thought to be because direct fecal markers suggest higher diagnostic ability in patients during clinical remission that less mucosal inflammation than the active phase. The optimal cutoff and AUCs for the ROC curves of LRG, PGE-MUM, and FC values for determining ER in this study were slightly lower than those reported in previous studies.^[15,18,24]^ One reason is that we focused solely on patients with UC in clinical remission only. Nevertheless, our study demonstrated the usefulness of these biomarkers. Despite these limitations, LRG and PGE-MUM are useful because of their accessibility and high accuracy.

Our results do not indicate that surveillance colonoscopy is unnecessary as colon cancer screening is still required. Approximately 30% of the patients with ER have histological inflammation; therefore, biopsy specimens are required to evaluate the mucosal conditions in addition to regular colonoscopy. In this study, approximately 40% of the patients with ER did not achieve HR. Although HR analysis was performed, no significant differences were observed, including FC levels, which is a useful marker for predicting MH.^[10]^ This is thought due to the inclusion of the patients during clinical remission that less mucosal inflammation than the active phase. This is likely due to the limited sample size and the consequent lack of statistical power for HR outcomes.

This study has several limitations, including the small sample size, the lack of healthy control and single-center design. Additionally, the evaluation of endoscopic and histological findings may be subject to human errors, the physical and environmental factors affecting LRG and PGE-MUM levels have not been examined, and the evaluation is based on a single measurement. Future investigations with a longer observation period and larger multicenter cohorts are warranted to confirm the usefulness of these biomarkers.

What should be performed if MH has not been achieved in clinical remission? Currently, there is no clear answer on how far treatment should be escalated. In patients in clinical remission with good quality of life, it is questionable whether treatment should be progressively escalated simply because MH has not been achieved. Escalating treatment may produce adverse effects and potentially lower the patient’s quality of life. Additionally, depending on the treatment, healthcare costs may increase. While the treatment goals for UC are widely recognized as clinical remission and MH, the definition of MH in UC remains controversial. In this study, less invasive biomarkers are useful for predicting MH, and that combining biomarkers allows for a more accurate diagnosis of MH in patients during remission. Although measuring multiple biomarkers may be challenging in clinical practice, this study could contribute to the future development of UC clinical practice.

In conclusion, LRG and PGE-MUM are potential biomarkers to confirm ER in patients with UC in clinical remission. Less invasive assessment using LRG and PGE-MUM combined with FC results in a more accurate diagnosis of ER even during remission.

## Author contributions

**Conceptualization:** Naohiro Kato, Ryusaku Kusunoki, Tsuyoshi Hatakeyama.

**Data curation:** Naohiro Kato, Ryusaku Kusunoki, Tsuyoshi Hatakeyama.

**Formal analysis:** Naohiro Kato, Ryusaku Kusunoki, Tsuyoshi Hatakeyama.

**Funding acquisition:** Naohiro Kato, Ryusaku Kusunoki, Toshio Kuwai.

**Investigation:** Naohiro Kato, Ryusaku Kusunoki, Tsuyoshi Hatakeyama.

**Methodology:** Naohiro Kato, Ryusaku Kusunoki.

**Project administration:** Naohiro Kato, Ryusaku Kusunoki, Toshio Kuwai.

**Resources:** Naohiro Kato, Ryusaku Kusunoki, Toshio Kuwai.

**Software:** Naohiro Kato, Ryusaku Kusunoki, Toshio Kuwai.

**Supervision:** Naohiro Kato, Ryusaku Kusunoki, Toshio Kuwai.

**Validation:** Naohiro Kato, Ryusaku Kusunoki, Toshio Kuwai.

**Visualization:** Naohiro Kato.

**Writing – original draft:** Naohiro Kato, Ryusaku Kusunoki.

**Writing – review & editing:** Ryusaku Kusunoki, Hiroki Kamada, Shigeaki Semba, Yuji Teraoka, Takeshi Mizumoto, Yuzuru Tamaru, Tsuyoshi Hatakeyama, Atsushi Yamaguchi, Hirotaka Kouno, Shigeto Yoshida, Toshio Kuwai.

## References

[1] Ng SC Shi HY Hamidi N. Worldwide incidence and prevalence of inflammatory bowel disease in the 21st century: a systematic review of population-based studies. Lancet. 2017;390:2769–78. 29050646 10.1016/S0140-6736(17)32448-0

[2] Danese S Fiocchi C. Fiocchi C: ulcerative colitis. N Engl J Med. 2011;365:1713–25. 22047562 10.1056/NEJMra1102942

[3] Turner D Ricciuto A Lewis A; International Organization for the Study of IBD. STRIDE-II: an update on the selecting therapeutic targets in inflammatory bowel disease (STRIDE) initiative of the international Organization for the Study of IBD (IOIBD): determining therapeutic goals for treat-to-target strategies in IBD. Gastroenterology. 2021;160:1570–83. 33359090 10.1053/j.gastro.2020.12.031

[4] Shah SC Colombel JF Sands BE Narula N. Mucosal healing is associated with improved long-term outcomes of patients with ulcerative colitis: a systematic review and meta-analysis. Clin Gastroenterol Hepatol. 2016;14:1245–55.e8. 26829025 10.1016/j.cgh.2016.01.015

[5] Yoon H Jangi S Dulai PS. Incremental benefit of achieving endoscopic and histologic remission in patients with ulcerative colitis: a systematic review and meta-analysis. Gastroenterology. 2020;159:1262–75.e7. 32585306 10.1053/j.gastro.2020.06.043 PMC7658293

[6] Yokoyama K Kobayashi K Mukae M Sada M Koizumi W. Clinical study of the relation between mucosal healing and long-term outcomes in ulcerative colitis. Gastroenterol Res Pract. 2013;2013:192794. 23762033 10.1155/2013/192794 PMC3665176

[7] Peyrin-Biroulet L Sandborn W Sands BE. Selecting therapeutic targets in inflammatory bowel disease (STRIDE): determining therapeutic goals for treat-to-target. Am J Gastroenterol. 2015;110:1324–38. 26303131 10.1038/ajg.2015.233

[8] Sands BE. Biomarkers of inflammation in inflammatory bowel disease. Gastroenterology. 2015;149:1275–85.e2. 26166315 10.1053/j.gastro.2015.07.003

[9] Nakarai A Kato J Hiraoka S. Evaluation of mucosal healing of ulcerative colitis by a quantitative fecal immunochemical test. Am J Gastroenterol. 2013;108:83–9. 23007005 10.1038/ajg.2012.315

[10] Cremer A Ku J Amininejad L. Variability of faecal calprotectin in inflammatory bowel disease patients: an observational case-control study. J Crohns Colitis. 2019;13:1372–9. 30944925 10.1093/ecco-jcc/jjz069

[11] Naka T Fujimoto M. LRG is a novel inflammatory marker clinically useful for the evaluation of disease activity in rheumatoid arthritis and inflammatory bowel disease. Immunol Med. 2018;41:62–7. 30938267 10.1080/13497413.2018.1481582

[12] Serada S Fujimoto M Terabe F. Serum leucine-rich alpha-2 glycoprotein is a disease activity biomarker in ulcerative colitis. Inflamm Bowel Dis. 2012;18:2169–79. 22374925 10.1002/ibd.22936

[13] Shinzaki S Matsuoka K Iijima H. Leucine-rich alpha-2 glycoprotein is a serum biomarker of mucosal healing in ulcerative colitis. J Crohns Colitis. 2017;11:84–91. 27466171 10.1093/ecco-jcc/jjw132 PMC5175492

[14] Yasutomi E Inokuchi T Hiraoka S. Leucine-rich alpha-2 glycoprotein as a marker of mucosal healing in inflammatory bowel disease. Sci Rep. 2021;11:11086. 34045529 10.1038/s41598-021-90441-x PMC8160157

[15] Shimoyama T Yamamoto T Yoshiyama S Nishikawa R Umegae S. Leucine-rich alpha-2 glycoprotein is a reliable serum biomarker for evaluating clinical and endoscopic disease activity in inflammatory bowel disease. Inflamm Bowel Dis. 2023;29:1399–408. 36334015 10.1093/ibd/izac230

[16] Arai Y Arihiro S Matsuura T. Prostaglandin E–major urinary metabolite as a reliable surrogate marker for mucosal inflammation in ulcerative colitis. Inflamm Bowel Dis. 2014;20:1208–16. 24846719 10.1097/MIB.0000000000000062

[17] Sakurai T Akita Y Miyashita H. Prostaglandin E–major urinary metabolite diagnoses mucosal healing in patients with ulcerative colitis in remission phase. J Gastroenterol Hepatol. 2022;37:847–54. 35064604 10.1111/jgh.15782 PMC9303914

[18] Ishida N Sugiura K Miyazu T. Prostaglandin E-major urinary metabolite predicts relapse in patients with ulcerative colitis in clinical remission. Clin Transl Gastroenterol. 2020;11:e00289. 33512810 10.14309/ctg.0000000000000289 PMC7732263

[19] Hagiwara SI Abe N Hosoi K. Utility of a rapid assay for prostaglandin E-major urinary metabolite as a biomarker in pediatric ulcerative colitis. Sci Rep. 2023;13:9898. 37336963 10.1038/s41598-023-37145-6 PMC10279732

[20] Geboes K Riddell R Ost A Jensfelt B Persson T Löfberg R. A reproducible grading scale for histological assessment of inflammation in ulcerative colitis. Gut. 2000;47:404–9. 10940279 10.1136/gut.47.3.404 PMC1728046

[21] Julious SA. Sample size of 12 per group rule of thumb for a pilot study. Pharm Stat. 2005;4:287–91.

[22] Billingham SA Whitehead AL Julious SA. An audit of sample sizes for pilot and feasibility trials being undertaken in the United Kingdom registered in the United Kingdom clinical research network database. BMC Med Res Methodol. 2013;13:104. 23961782 10.1186/1471-2288-13-104 PMC3765378

[23] Kim DJ Jeoun YM Lee DW Koo JS Lee SW. Usefulness of fecal immunochemical test and fecal calprotectin for detection of active ulcerative colitis. Intest Res. 2018;16:563–70. 30301335 10.5217/ir.2018.00020 PMC6223456

[24] Takenaka K Kitazume Y Kawamoto A. Serum leucine-rich alpha-2 glycoprotein: a novel biomarker for transmural inflammation in Crohn’s disease. Am J Gastroenterol. 2022;118:1028–35. 36571769 10.14309/ajg.0000000000002127

